# The Pace of Prostatic Intraepithelial Neoplasia Development Is Determined by the Timing of *Pten* Tumor Suppressor Gene Excision

**DOI:** 10.1371/journal.pone.0003940

**Published:** 2008-12-15

**Authors:** H. Artee Luchman, Hallgrimur Benediktsson, Michelle L. Villemaire, Alan C. Peterson, Frank R. Jirik

**Affiliations:** 1 Department of Biochemistry and Molecular Biology, and The McCaig Institute for Bone and Joint Health, University of Calgary, Calgary, Alberta, Canada; 2 Department of Pathology and Laboratory Medicine, University of Calgary, Calgary, Alberta, Canada; 3 Royal Victoria Hospital, Department of Neurology and Neurosurgery, McGill University, Montreal, Quebec, Canada; Universität Heidelberg, Germany

## Abstract

Loss of the PTEN tumor suppressor is a common occurrence in human prostate cancer, particularly in advanced disease. In keeping with its role as a pivotal upstream regulator of the phosphatidylinositol 3-kinase signaling pathway, experimentally-induced deletion of *Pten* in the murine prostate invariably results in neoplasia. However, and unlike humans where prostate tumorigenesis likely evolves over decades, disease progression in the constitutively *Pten* deficient mouse prostate is relatively rapid, culminating in invasive cancer within several weeks post-puberty. Given that the prostate undergoes rapid androgen-dependent growth at puberty, and that *Pten* excisions during this time might be especially tumorigenic, we hypothesized that delaying prostate-specific *Pten* deletions until immediately after puberty might alter the pace of tumorigenesis. To this end we generated mice with a tamoxifen-inducible Cre recombinase transgene enabling temporal control over prostate-specific gene alterations. This line was then interbred with mice carrying floxed *Pten* alleles. Despite evidence of increased Akt/mTOR/S6K axis activity at early time points in *Pten*-deficient epithelial cells, excisions induced in the post-pubertal (6 wk-old) prostate yielded gradual acquisition of a range of lesions. These progressed from pre-malignant changes (nuclear atypia, focal hyperplasia) and low grade prostatic intraepithelial neoplasia (PIN) at 16–20 wks post-tamoxifen exposure, to overtly malignant lesions by ∼1 yr of age, characterized by high-grade PIN and microinvasive carcinoma. In contrast, when *Pten* excisions were triggered in the pre-pubertal (2 week-old) prostate, neoplasia evolved over a more abbreviated time-frame, with a spectrum of premalignant lesions, as well as overt PIN and microinvasive carcinoma by 10–12 wks post-tamoxifen exposure. These results indicate that the developmental stage at which *Pten* deletions are induced dictates the pace of PIN development.

## Introduction

Genetic alterations in a variety of different oncogenes and tumor suppressor genes have been associated with human prostate tumorigenesis (reviewed in [1]; [2]). Of these, mutations involving the *PTEN* (phosphatase and tensin homolog deleted on chromosome 10) tumor suppressor are amongst the most commonly encountered, with loss of function mutations being reported in ∼30% of primary cancers, and in more than 60% of metastases (reviewed in [3]). Echoing these findings, deletion of *Pten* in the developing murine prostate leads to early onset and rapidly progressive neoplasia [4]–[8]. PTEN's importance lies primarily in its ability to regulate the levels of membrane PI(3,4,5)P3 (PIP3) generated by the actions of phosphatidylinositol 3′-kinase (PI3K) (reviewed in [9]). PTEN dephosphorylates PIP3, yielding PI(4,5)P2, thus PI3K activity (for example, in response to receptor protein tyrosine kinase activation) in Pten-deficient cells results in higher and more sustained levels of PIP3. PIP3-dependent pathways, in turn, regulate various cellular processes, including, rate of protein translation, susceptibility to apoptosis and anoikis, entry into the cell cycle, differentiation, and motility (reviewed in [9]). Key effectors lying downstream of PIP3 that promote tumorigenesis include such molecules as PDK1, Akt/protein kinase B (PKB), and the two mammalian target of rapamycin-containing complexes, mTOR1 and mTOR2 [10], [11]. The PI3K/AKT/mTOR pathway in particular often plays a fundamental role in supporting cancer cell metabolism, growth, and survival [12].

The ability to manipulate the mouse genome has allowed the evaluation of genetic alterations potentially involved in human prostate tumorigenesis, as well as the identification and preclinical validation of molecular targets for potential pharmacological intervention [13]. In the case of *PTEN*, loss of the cognate gene in the murine prostate has been shown to trigger rapid onset of disease, with prostatic intraepithelial neoplasia (PIN) and invasive adenocarcinomas ensuing within a few weeks of puberty [4], [14]–[17]; reviewed in [18]). Even taking into account the relatively short lifespan of mice, such a rapid evolution of disease is not reflective of disease progression in humans, that likely takes place over decades as a consequence of an accumulation of genetic changes and where intraepithelial lesions tend only to be observed beyond middle age. Indeed, most cases of prostate cancer are diagnosed in men over the age of 65 [19]. Thus far there has been a paucity of mouse models of prostate cancer able to clearly delineate the various pathological grades seen in the human disease, from the earliest premalignant changes to overt invasive carcinoma and metastasis [20].

There has been a need for mouse models that better mimic the slow progression to cancer typical of human prostate tumorigenesis. One of the steps towards attaining this goal has been the development of inducible systems that allow genetic alterations to be confined to the prostatic epithelium of the mature gland, the target tissue involved in the acquisition of genetic alterations over the span of a human lifetime. To this end, we generated a murine line, ARR2PB-CreER(T2), expressing a 4-hydroxy-tamoxifen (OHT)-inducible Cre recombinase, Cre-ER(T2), under the control of the ARR2-rat probasin composite promoter [21]. This novel system allows temporal control over the timing of gene deletions in the prostatic epithelium.

Since the prostate epithelium undergoes rapid growth at puberty, we hypothesized that this might account for the rapid progression of disease in mice expressing constitutive Cre-triggered deletions of *Pten*. Supporting this idea, we found that OHT-induced Cre-mediated *Pten* excisions in the gland after puberty led to the very gradual development of a range of premalignant lesions. Over the course of a year these mice went on to develop high-grade PIN lesions as well as invasive carcinoma. The delayed latencies occurred despite evidence of prominent activation of the pro-tumorigenic Akt/mTOR/S6K pathway at all stages of the disease. In support of the hypothesis that the timing of *Pten* loss is an important variable in mouse prostate tumorigenesis, *Pten* excisions triggered in the pre-pubertal prostate accelerated the progression to PIN and microinvasive carcinoma.

## Results

### Prostate histopathology in OHT-treated *ARR2PBCreER(T2)×Pten^fl/fl^* mice

**Figure 1:**
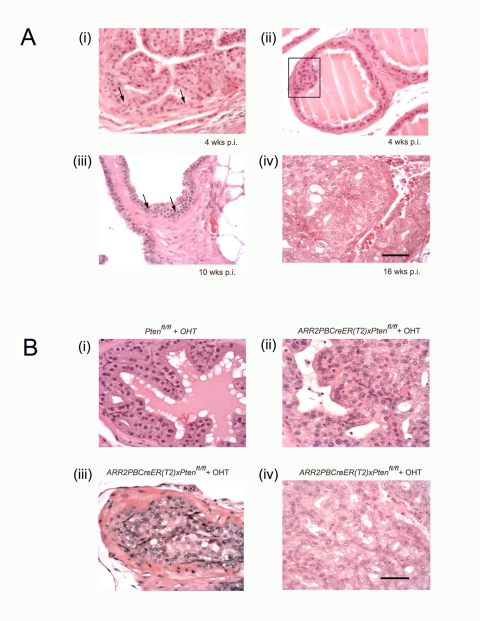
Transition from the earliest stages of transformation to occur progressively in *ARR2PBCreER(T2)×Pten^fl/fl^* mice following OHT exposure. (A) Nuclear enlargement with prominent and multiple nucleoli is seen in the luminal epithelial layer at different times post-OHT; in single cells at 4 wks post-OHT (arrows (i)); becoming more widespread at 8–10 wks post-OHT (arrows (iii)) and in the majority of cells in PIN lesions at 16–20 wks post-OHT (iv). Low grade hyperplasia is seen in the luminal layer at 4 wks post-OHT (rectangle (ii)). At 10 wks post-OHT, hyperplasia became more pronounced and nuclear enlargement with prominent nucleoli and hyperchromasia became more prevalent (arrows (iii)). Early stages of PIN, such as nuclear overlapping and mild tufting are noticeable by 10 wks post-OHT (iii). At 16–20 wks post-OHT more advanced PIN stages were evident (iv), with overt tubular dysplasia while basement membranes remained unbreached. PIN was prominent and most cells within these lesions showed nuclear enlargement, prominent nucleoli, hyperchromasia, and abnormal morphology (iv). (B) High grade PIN lesions were present in mice at 16–20 wks post-OHT. (i) While *Pten^fl/fl^* controls showed normal morphology after OHT treatment, PIN lesions are seen 16–20 wks post-OHT administration in *ARR2PBCreER(T2)×Pten^fl/fl^* mice, with different categories of high grade PIN being evident; including as tufting, micropapillary and flat atypia (ii), and cribriform structures (iii) and (iv) were seen in both focal intra-tubular as well as the more widespread lesions in the experimental animals. Scale bars: 50 µm.

As *Pten* gene deletions in the prostate have been shown to lead to rapid onset of tumorigenesis, we investigated the effects of delaying *Pten* excisions until after the gland had developed. Thus, *ARR2PBCreER(T2)×Pten^fl/fl^* or *Pten^fl/fl^* control mice were injected with OHT daily for 5 consecutive days starting at 6 wks of age and then sacrificed at either 4–10,16–20, or 30–40 wks p.i. In the 4–10 wks p.i. group, *ARR2PBCreER(T2)×Pten^fl/fl^* mice treated with OHT demonstrated nuclear atypia and increased prominence of nucleoli in sporadic cells within the prostatic epithelium (arrows - **Figure 1A**(**i**) and (**iii**)), as well as early hyperplastic lesions at 4-wks post-OHT mice (rectangle- **Figure 1A**(**ii**)) with these being more obvious at 10-wks post-OHT mice (**Figure 1A**(**iii**)). At 16–20 wks p.i. the premalignant phenotype became much more evident, such that experimental animals displayed increased cellular size and nuclear atypia, as well as abnormal cellular morphology of luminal epithelial cells (**Figure 1A**(**iv**)). Of the 13 experimental animals, 11 contained focal areas with hyperplastic lesions ranging from mild to pronounced, and 6 out the mice exhibited advanced PIN lesions (**Figure 1B**(**ii–iv**) and **Table 1**). Control animals did not display these phenotypic changes (**Figure 1B**(**i**)) except perhaps for two mice with mild focal thickening of the luminal epithelium likely due to hyperplasia (**Table 1**). There was no additional progression past PIN lesions in 4 experimental animals when these were aged to 30–40 wks p.i., however, the PIN lesions became more prominent, and showed a wider area of distribution within the prostate (data not shown). Furthermore, none of the non-OHT exposed *ARR2PBCreER(T2)×Pten^fl/fl^*, control *Pten^fl/fl^*, or the *ARR2PBCreER(T2)* mice developed any evidence of premalignant lesions in their prostates (data not shown).

**Table 1:** Morphologic alterations in the prostates of *ARR2PBCreER(T2)*×*Pten^fl/fl^* mice injected at 6 wks of age, and subjected to histopathological analysis 16–20 wks post-OHT exposure.

Mouse Genotype	Abnormal cellular morphology^1^	Atypical nuclear morphology^2^ and increased number of nucleoli	Focal Hyperplasia	PIN lesions
Control *Pten^fl/fl^*	None of 9	None of 9	Very small in 2 of 9	None of 9
Experimental *ARR2PBCreER(T2)×Pten^fl/fl^*	10 of 13	13 of 13	11 of 13	6 of 13

1 Abnormal cellular morphology refers to nuclear enlargement, prominent nucleoli, hyperchromasia, and deviations in cell size and appearance.

2 Atypical nuclear morphology refers to nuclei displaying nuclear and nucleolar enlargement and increased number of nucleoli.

### Increased proliferation in the epithelium of OHT-treated *ARR2PBCreER(T2)×Pten^fl/fl^* mice

**Figure 2:**
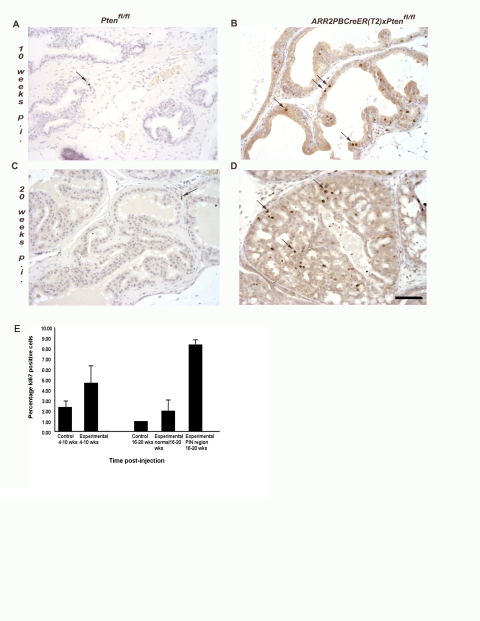
There is increased cell proliferation in the luminal epithelia of *ARR2PBCreER(T2)×Pten^fl/fl^* mice exposed to OHT. Immunohistochemical analyses revealed increased numbers of proliferating cells within the luminal epithelium of *ARR2PBCreER(T2)×Pten^fl/fl^* mice treated with OHT at 6 wks of age as indicated by Ki67 staining, as shown by the dark dots and arrows in (B) and (D). (A) and (C) control *Pten^fl/fl^* at 10 wks and 20 wks post-OHT; (B) *ARR2PBCreER(T2)×Pten^fl/fl^* at 10 wks post-OHT and a (D) a representative PIN lesion in a *ARR2PBCreER(T2)×Pten^fl/fl^* mouse at 20 wks post-OHT. Scale bar: 50 µm. (E) Quantification of the percentage of proliferating cells in control and *ARR2PBCreER(T2)×Pten^fl/fl^* aged for 4–10 wks and 16–20 wks post-OHT injections. There is a significant increase in the percentage of proliferating cells in the PIN regions of 16–20 p.i. experimental animals both over control animals and the normal regions within the experimental animals (p-value<0.001).

The Ki67 marker was used to show the level of proliferation in the prostatic epithelium of OHT-treated *ARR2PBCreER(T2)×Pten^fl/fl^* mice. At 4–10 wks post-OHT treatment, Cre-positive animals demonstrated increased numbers of luminal epithelial cells positive for Ki67 (**Figure 2B**). This effect was much more pronounced at 16–20 wks post-OHT and as would be predicted, was especially striking in the hyperplastic and PIN lesions (**Figure 2D**). In contrast, control *Pten^fl/fl^* prostates treated with OHT contained few, if any, Ki67-positive cells (**Figure 2A and C**). These findings demonstrated that loss of Pten was accompanied by a progressive increase in epithelial cell proliferation as shown by quantification of Ki67-positive cells at 4–10 wks and 16–20 wks (**Figure 2E** **)**.

### PIN lesions of *ARR2PBCreER(T2)×Pten^fl/fl^* OHT-exposed mice demonstrate Pten deficiency

**Figure 3:**
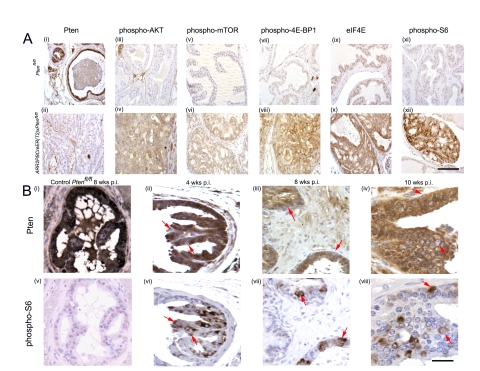
Downstream components of the PI3K signaling pathway are activated in PIN lesions of *ARR2PBCreERT2*×*Pten^fl/fl^* mice post-OHT exposure. (A) Immunohistochemistry showing decreased Pten levels in a representative PIN lesion ((ii) bottom panel); top panel (i) shows Pten levels in prostate epithelium of control *Pten^fl/fl^* mice. Immunohistochemistry revealed increased levels of phospho-AKT, phospho-mTOR, phospho-4E-BP1, eIF4E, and phospho-S6 (bottom panels (iv); (vi); (viii); (x); (xii) respectively) in PIN regions of positive animals (bottom panels) as compared to controls (top panels (iii); (v); (vii); (ix); (xi)). (B) Pten loss in the prostatic epithelium is associated with increase in pS6 ribosomal protein levels in the preneoplastic phase of *ARR2PBCreER(T2)×Pten^fl/fl^* mice post-OHT. Serial sections stained with either anti-Pten or anti-pS6 antibodies showed that loss of Pten could be correlated with increased phospho-S6 signal and that this could be detected at the single cell level during early time points post-OHT (arrows), even at a time when cellular and nuclear atypia were not present; as indicated by the red arrows - (ii) and (vi) 4 wks; (iii) and (vii) 8 wks; (iv) and (viii) 10 wks post-OHT. (i) and (v) show Pten, and phospho-S6 immunoreactivity, respectively, in serial sections form the prostate of a *Pten^fl/fl^* control mouse 8 wks post-OHT. Scale bars: A (i–xii), 100 µm; B (i–vi), 37.5 µm.

When compared to the levels of Pten in the luminal epithelium of control *Pten^fl/fl^* animals (**Figure 3A**(**i**)), Pten protein deficiency was evident in PIN lesions of *ARR2PBCreER(T2)×Pten^fl/fl^* mice at 16–20 wks OHT p.i. (**Figure 3A**(**ii**)). At 4–10 wks p.i., Pten deficiency was seen in a small number of cells (arrows **Figure 3B**(**i–ii**)), and loss of Pten immunoreactivity was particularly evident in hyperplastic regions (arrows **Figure 3B**(**iii**)). These results were consistent with Pten deficiency being present in a subset of cells that together with increased proliferation and additional genetic events gradually evolved into the PIN lesions observed in the older mice. As only a few cells in a given tubule tended to show loss of Pten immunoreactivity at 4–10 wks post-OHT exposure, it suggested that Pten excisions at the epithelial stem cell level may have been infrequent.

### Evidence for PI3K pathway activation in PIN lesions of *ARR2PBCreER(T2)×Pten^fl/fl^* mice 16–20 wks post-OHT treatment

To assess PI3K pathway activity components known to be affected by the loss of Pten were examined by immunohistochemistry of paraffin sections from mice 16–20 wks post-OHT exposure, these included: AKT, mTOR, 4E-BP1, eIF4E and S6. Levels of phospho-AKT and phospho-mTOR were upregulated in the PIN lesions of OHT-treated *ARR2PBCreER(T2)×Pten^fl/fl^* mice (**Figure 3A**(**iv**,**vi**)). The levels of phospho-4E-BP1, eIF4E, and phospho-S6-ribosomal protein immunoreactivity were also upregulated in the PIN lesions (**Figure 3A**(**viii**,**x**,**xii**)), indicating that the AKT/mTOR/S6K axis was activated in the prostatic lesions. This feature, obvious in PIN lesions as these contained large numbers of Pten deficient cells (**Figure 3A**(**ii**)), was also observed in hyperplastic regions of 16–20 wks post-OHT treated experimental mice and even in isolated tubular cells in mice at 4–10 wks post-OHT treatment (data not shown).

### Direct correspondence between Pten deficiency and phospho-S6 ribosomal protein upregulation in pre-neoplastic epithelial cell lesions

Pten-deficient cell numbers were higher in the juxta-luminal epithelial regions of *ARR2PBCreER(T2)×Pten^fl/fl^* prostates post-OHT treatment and these were associated with cellular and nuclear atypia, as well as hyperplastic regions and PIN (**Figure 3A**(**ii**); **Figure 3B**(**i–iii**)). By performing immunohistochemistry for Pten and phospho-S6 ribosomal protein on sequential sections, we found that cells and regions lacking Pten also expressed high levels of phospho-S6, even at 4–10 wks p.i. This phenomenon was even evident within single cells at the earliest time points (arrows **Figure 3B**(**i,ii,iv,v**)), becoming more dramatic within Pten-deficient cells contained within hyperplastic regions (arrows **Figure 3B**(**iii,vi**)). These results demonstrated that there was a direct correlation between Pten-deficiency and increased S6 kinase activity, and that this feature was present early on, well before the appearance of histologically evident cellular and nuclear atypia, or hyperplasia.

### Progression to carcinoma in *ARR2PBCreER(T2)×Pten^fl/fl^* mice 42–52 wks post-OHT

**Figure 4:**
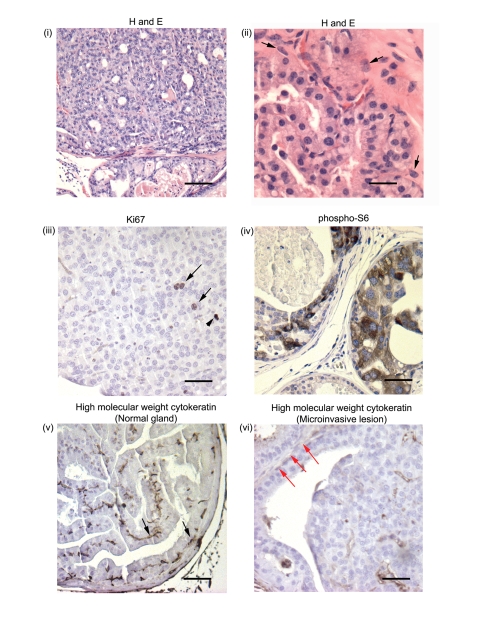
Transition from PIN to invasive carcinoma is seen in *ARR2PBCreER(T2)×Pten^fl/fl^* mice exposed to OHT and aged for 42–52 wks. (i) Widespread high grade PIN lesions were seen in multiple glands within the prostates of experimental animals at 42–52 wks post-OHT, (ii) with abnormal cellular and nuclear morphology and microinvasive lesions (arrows). (iii) Proliferating cells within these lesions were detected by immunostaining with the anti-Ki67 antibody (arrows; arrowhead shows a mitotic figure) and high levels of phospho-S6 expression were also detected (iv). Representative immunohistochemical analysis using an antibody against high molecular cytokeratins 1,5,10, and 14 that stains basal cells: although basal cytokeratin staining was present within normal-appearing glands (v) (black arrows), with progression to invasive carcinoma there was a lack of cytokeratin staining (vi) (red arrows) within the same prostate. Scale bars: (i), 100 µm; (ii–vi), 25 µm.

Progression from PIN to invasive carcinomas was seen in experimental animals at 42–52 wks post-OHT administration. We found that the area composed of PIN lesions increased greatly with aging, with multiple glands developing PIN (**Figure 4** **(i and ii)**). There were proliferating cells within these lesions as shown by immunostaining with the Ki67 antibody (**Figure 4** (**iii**) arrows) and high levels of phospho-S6 expression (**Figure 4** (**iv**)), indicating that pAKT/pmTOR/pS6 pathway had remained active. Immunohistochemistry for cytokeratins 1, 5, 10 and 14 (clone 34B12 from Dako), that stains basal cells, showed that while normal glands within the same animal maintained basal cell cytokeratin staining (arrows **Figure 4** (**v**)), the PIN lesions in the animals aged for 42–52 wks aged animals had progressed to carcinoma as suggested by the lack of cytokeratin staining for basal cells (red arrows **Figure 4** (**vi**)).

### Accelerated progression to PIN and microinvasive carcinoma when *Pten* is deleted in the prostates of 2 wk-old *ARR2PBCreER(T2)×Pten^fl/fl^* mice

**Figure 5:**
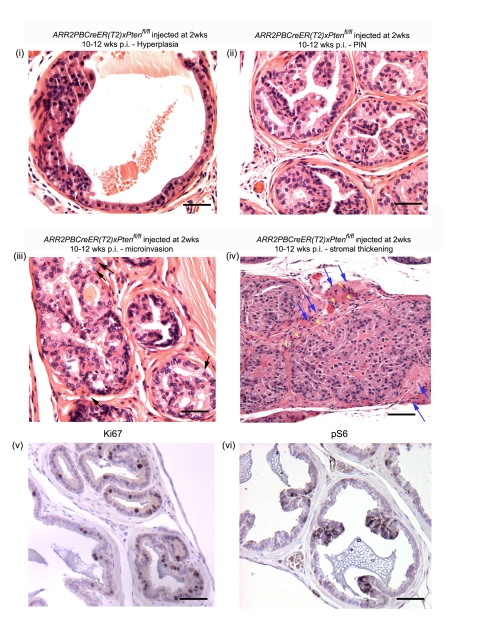
Histological analysis of *ARR2PBCreER(T2)×Pten^fl/fl^* mice exposed to OHT at 2 wks of age. *ARR2PBCreER(T2)×Pten^fl/fl^* injected at 2 wks of age displayed a wide range of lesions by 10–12 wks post-OHT: (i) hyperplastic lesion, (ii) PIN, (iii) high grade PIN with occasional microinvasive cells (arrows), (iv) high grade PIN lesions with a wide distribution area, stromal invasion of epithelial cells (yellow arrowheads) and displaying stromal thickening (blue arrows), (v) increased proliferation in a positive mouse aged for 6 wks as indicated by Ki67 staining, (vi) positive phospho-S6 staining of a positive animal at 6 wks post-OHT). Scale bars: (i–iii and v–vi), 50 µm; (iv), 100 µm.

A wide range of lesions was seen in the prostates of mice that had been injected with OHT at 2 wks of age and then aged for 4–6 wks and 10–12 wks (**Figure 5** and **Table 2**). Nuclear atypia and abnormal cellular morphology, as well as hyperplastic regions (**Figure 5**(**i**)) and low grade PIN lesions (**Figure 5** (**ii**)) were already evident in animals aged for 4–6 wks p.i. Importantly, at 10–12 wks p.i. the phenotype became much more pronounced such that all of the experimental animals displayed increased cell size and nuclear atypia, abnormal cellular morphology of luminal epithelial cells, hyperplastic lesions, and high grade PIN (**Figure 5** **(iii–iv)**). Two of the 6 experimental animals in this age group had even developed microinvasive carcinoma (arrows in **Figure 5**(**iii**)). Stromal thickening, suggestive of an invasive phenotype, was noticeable in animals at 10–12 weeks p.i. (blue arrows **Figure 5** (**vi**)). At both 4–6 wks and 10–12 wks p.i., Cre-positive animals were found to have increased numbers of luminal epithelial cells positive for the proliferation marker Ki67 (**Figure 5** (**v**) shows a representative experimental animal at 4–6 wks p.i) and were also positive for phospho-S6 (**Figure 5** (**vi**) shows a representative experimental animal at 4–6 wks p.i).

**Table 2:** Morphologic alterations in the prostates of *ARR2PBCreER(T2)*×*Pten^fl/fl^* mice injected at 2 wks of age, and subjected to histopathological analysis 10–12 wks post-OHT exposure.

Mouse Genotype	Abnormal cellular morphology^1^	Atypical nuclear morphology^2^ and increased number of nucleoli	Focal Hyperplasia	PIN lesions	Microinvasive carcinoma
Control *Pten^fl/fl^*	0/4	0/4	0/4	0/4	0/4
Experimental *ARR2PBCreER(T2)*×*Pten^fl/fl^*	6/6	6/6	6/6	6/6	2/6

1 Abnormal cellular morphology refers to nuclear enlargement, prominent nucleoli, hyperchromasia, and deviations in cell size and appearance.

2 Atypical nuclear morphology refers to nuclei displaying nuclear and nucleolar enlargement and increased number of nucleoli.

### Increased apoptosis observed in prostatic epithelium following *Pten* gene deletion

**Figure 6:**
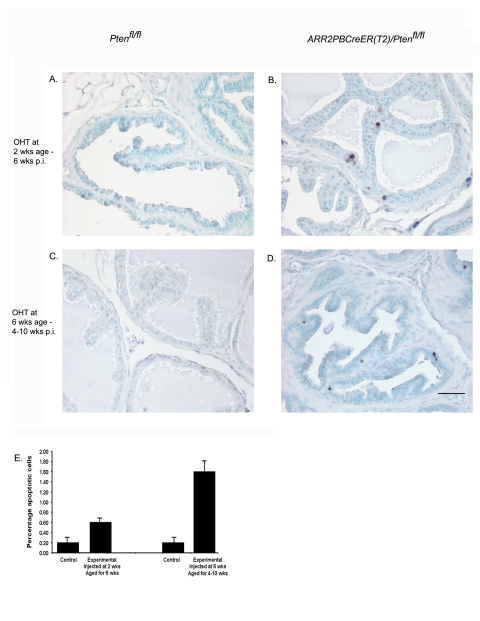
There is increased cell death in the luminal epithelia of *ARR2PBCreER(T2)×Pten^fl/fl^* mice exposed to OHT. There were increased numbers of apoptotic cells within the luminal epithelium of *ARR2PBCreER(T2)×Pten^fl/fl^* mice treated with OHT at 2 wks of age and aged for 6 wks (B) and also in *ARR2PBCreER(T2)×Pten^fl/fl^* mice treated with OHT and aged for 4–10 wks (D). (E) Quantification of the percentage of apoptotic cells in control and *ARR2PBCreER(T2)×Pten^fl/fl^* mice. There was a significant increase in the percentage of apoptotic cells over controls in *ARR2PBCreER(T2)×Pten^fl/fl^* injected with OHT either at 2 wks and 6 wks of age (p-value<0.001). Scale bar: 50 µm.

TUNEL assay was used to show the level of apoptotic cells in the prostatic epithelium of *ARR2PBCreER(T2)×Pten^fl/fl^* mice previously treated with OHT either at 2 wks and then aged for 6 wks p.i. (**Fig. 6** (**B**)), or treated with OHT at 6 wks and then aged for 4–10 wks p.i (**Fig. 6** (**D**)). In both groups, Cre-positive animals revealed increased numbers of apoptotic luminal epithelial cells as compared to controls (p-value<0.001) as shown by the quantification of TUNEL-positive cells (**Figure 6E**). These results showed that loss of Pten was accompanied by a significant increase in epithelial cell death.

## Discussion

Prostate-specific promoters such as the composite probasin promoter used herein have previously been employed to generate a wide variety of transgenic lines [17], [22]–[24]; reviewed in [18]. These have enabled evaluation of the consequences of prostate-specific expression of genes implicated in human prostate cancer. Germline knockout models, where all cells in the animal harbor the genetic alteration, have also been used to study prostate tumorigenesis (reviewed in [18]). However, owing to the lack of cell-type specific deletion in the latter, it has been difficult to attribute the resulting phenotypes solely to a defect within the prostatic epithelium. Thus, in the last few years, conditional gene alterations have been used to investigate the ability of specific mutations to promote the development of prostate neoplasia [17], [25] (reviewed in [18]).

Various models of prostate cancer have shown that mutations in tumor suppressors, or activation of oncogenes during androgen-induced gland development at puberty can lead to prostate cancer. In such models, however, tumor progression often occurs at an accelerated pace with progression to adenocarcinomas and invasive carcinomas being evident within a few weeks after puberty [4], [14]–[17]. For example, the *PBCre4×Pten^flox/flox^* prostate cancer model [17]; (reviewed in [18]) has been shown to mimic human prostate cancer development, including progression from hyperplasia to PIN, to adenocarcinoma and metastasis. As such, this appears to be one of the few murine models that replicates the spectrum of grades and stages observed in human prostate cancer [18]. However, the age at which invasive tumors are observed in this model would correspond to early adulthood in humans and do not accurately reflect the more protracted developmental time-course of prostate cancer in humans.

Conditional mutagenesis systems permitting external control over the timing of genetic alterations are attractive as they allow phenotypic analyses of mutations in the context of the mature, or even aged, prostate gland. The inducible transgenic mouse model we previously developed [21] has allowed us to induce prostate luminal epithelial cell-specific gene excisions in a temporally controlled manner. This is a valuable feature given that genetic and epigenetic alterations accumulated by the mature gland likely play a role in driving progression of prostate cancer in humans. Indeed, we found that *Pten* excisions in prostates of 6 wk-old *ARR2PBCreER(T2)×Pten^fl/fl^* resulted in a very gradual progression to PIN and invasive carcinoma. Our findings are in agreement with the recently published results of Ratnacaram et al., [26] who showed that the CreER(T2) expressed under the control of the PSA (prostate specific antigen) promoter yielded a similarly slowed pace of disease progression when crossed onto the *Pten^fl/fl^* background. In our experiments, where we used 10–fold less OHT (0.1 mg per day) than Ratnacaram et al., [26] (1 mg per day), to activate CreER(T2) over a one-week period, we obtained a very similar pattern of disease progression stemming from *Pten* deletion in the adult animals. These two sets of results, using two different prostate-specific promoter transgene systems to drive CreER(T2) recombinase expression, support the idea that the developmental stage at which *Pten* is excised can affect the pace of prostate tumorigenesis.

We have shown that *Pten* deletions, in the mature prostate leads to a wide range of pre-malignant lesions, and as illustrated by Figure 1, even very early lesions could be resolved. We also obtained immunohistochemical evidence of cell signaling aberrations, consistent with the loss of Pten, and these were discernable early on (Figure 3B) within isolated populations of epithelial cells. For example, Pten loss was accompanied by increased pS6 levels (Figure 3B(iv–vi)) even prior to any morphological signs of atypia. Thus, although loss of the Pten tumor suppressor and increased PI3K pathway signaling is a key initiating factor in tumorigenesis, it appears that additional cytogenetic or epigenetic events must occur prior to the development of the PIN lesions as observed at later stages in our mice (Figure 1B). Interestingly, we did not observe progression past high grade PIN lesions until mice had been aged for almost a year post-OHT administration (Figure 4). The nature of the acquired genetic events required to promote tumor progression in our model remain to be elucidated. The application of new genomics and proteomics techniques to our model, with its gradual stepwise development of pathological lesions, could be useful in identifying sequences of alterations of potential relevance to human prostate cancer, and in particular, cancers featuring the loss of PTEN.

In contrast to the results obtained when *Pten* deletion was triggered by OHT in mice at 6 wks of age, *Pten* excisions induced immediately prior to the onset of puberty led to more rapid pace of disease progression, with PIN lesions already being present at 4–6 wks post-OHT (Figure 5), and high grade PIN lesions and microinvasive carcinoma developing at 10–12 wks post-OHT (Figure 5). This result was striking given that progression past PIN was only seen at 42–52 wks post-OHT when *Pten* excisions were triggered at 6 wks of age. For the purposes of comparison, we also established the constitutive *PBCre4×Pten^flox/flox^* model that progresses rapidly from PIN to adenocarcinoma and metastases. In our colony, disease progression in these mice was modestly delayed (by a few weeks) as compared to what was reported by Wang et al, [4](Table 3), the *PBCre4×Pten^flox/flox^* mice developed invasive carcinomas and adenocarcinomas at a time when the *ARR2PBCreER(T2)×Pten^fl/fl^* mice treated with OHT at 2 wks of age, were starting to develop microinvasive carcinomas. Thus, in our model, excisions of *Pten* induced at 2 wks of age more closely resembled the phenotype observed in *PBCre4×Pten^flox/flox^* mice, while delaying *Pten* excision until after puberty greatly slowed the pace of disease development (Table 3). The importance of the timing of *Pten* deletions was also highlighted by Backman et al. [5] using the *MMTVCre×Pten^fl/fl^* mouse model. In this case, early *Pten* deletions led to epithelial hyperplasia as early as 5 days of age and high-grade PIN and prostate carcinoma by 2 wks of age.

**Table 3:** Timelines of disease progression in the *PbCre4×Pten^fl/fl^* and *ARR2PBCreER(T2 )×Pten^fl/fl^* mice injected with OHT either at two weeks or six weeks.

	Hyperplasia	PIN	Invasive Carcinoma	Metastases
*PBCre4×Pten^fl/fl^*	4–6 wks of age	PIN by 4–6 wks of age – high grade PIN 7–9 wks of age	10+ wks of age	25+ wks of age in lymph nodes in small number of animals
*ARR2PBCreER(T2)×Pten^fl/fl^* injected with OHT at 2 wks of age	4–6 wks p.i.	PIN by 4–6 wks p.i. – high grade PIN 10–12 wks p.i.	10–12 wks p.i.	Not seen as of 25 wks p.i.
*ARR2PBCreER(T2)×Pten^fl/fl^* injected with OHT at 6 wks of age	4–10 wks p.i.	16–20 wks p.i	52+ wks p.i.	Not seen as of 52+ wks p.i.

What accounted for the more aggressive disease course when *Pten* excisions were induced prior to gland maturation? Growth factors and other signals that are able to drive the PI3K pathway may differ between the juvenile and adult glands. For example, fibroblast growth factor 10 expression is prominent in the mesenchyme surrounding the developing prostate but is then switched off once the gland matures [27]. Furthermore, and suggesting a mechanism whereby Pten deletion in the developing gland might be especially tumorigenic, it was recently shown using an *in vivo* epithelial-stromal reconstitution system that enhanced mesenchymal expression of FGF10 led to the formation of multifocal PIN lesions in mice [28]. Also, the loss of *Pten* just prior to puberty, a time when epithelial proliferation is much more prominent that in the mature gland, could mean that more cells are placed at risk of transformation as a result of the chromosomal instability that has been proposed to accompany loss of Pten expression (reviewed in [29]).

While *Pten* excision obviously initiates the progression from hyperplasia to PIN in our mice, lack of Pten by itself, especially in the mature prostate, was not sufficient to generate the more advanced lesions typical of the constitutively active Cre models of *Pten* excision. While this might reflect excision frequencies within the gland, it could also be due to an intrinsic difference between the developing versus the mature gland that determines how the cells respond to *Pten* loss. For example, it has been suggested that of the lack of PTEN can be detrimental to tumour growth in the absence of other mutations (reviewed in [29]), and complete loss of this tumour suppressor can promote a senescence response that opposes tumour progression [30]. It was found that while *Trp53* inactivation in the mouse prostate failed to produce neoplasia, and *Pten* inactivation produced non-lethal invasive prostate cancer after a long latency, the combined inactivation of *Trp53* and *Pten* in the prostate elicited invasive prostate cancer in mice as young as two weeks of age. In this case, *Pten* inactivation was found to trigger growth arrest through a p53-dependent cellular senescence response that was ‘rescued’ by the combined loss of *Pten* and *Trp53* [30]. It was also shown that *Pten* inactivation in the prostate correlated with an increased proliferation rate of the epithelial cells, but not with a reduced level of apoptosis [6]. This suggested that while *Pten* inactivation promotes tumorigenesis there are also mechanisms at play which may push the Pten-deficient cells towards apoptosis. Although we found evidence of increased apoptosis within our experimental animals treated either at 2 wks or 6 wks of age (Figure 6), mice injected at 2 wks still progressed to PINs and microinvasive cancer more rapidly suggesting that the delayed latencies seen following *Pten* excision at 6 wks of age might have been a consequence of tumorigenic changes being targeted to a residual population of cells that had evaded the Pten loss-induced senescence response. Lastly, it is possible that the prostate progenitors responsible for the rapid pubertal growth of the gland are less susceptible to the senescence-promoting effect of Pten deficiency.

Using the *PBCre4×Pten^flox/flox^* model, Wang et al., [4] showed that, *Pten* loss can lead to increased stem/progenitor cell proliferation, which in turn may be associated with prostate tumor initiation and progression. However, the authors did not rule out the possibility that Pten loss might also be able to promote the transformation of cells with limited replicative capacity, providing them with self-renewal potential. In our model, *Pten* deletion in the mature glad did not result in early invasive adenocarcinomas and metastases, suggesting that loss of Pten within a differentiating population with low proliferative potential might only have a limited ability to augment residual proliferative capacity. In the adult prostate the number of self-renewing cells would be smaller than in the developing prostate, and this could be a factor in the slower pace of tumor progression seen in our model. Wang et al., [4] proposed that *Pten* loss may either increase the pool of stem and early progenitor cells, or that it might be a transforming event in non-actively dividing cells, leading them to acquire self-renewing proliferative potential. Since we did not observe rapid progression to invasive carcinoma and have not as yet seen metastases, it is possible that in our model *Pten* deletions are confined to cells with more limited self-renewal capacity. This observation was reinforced by the results of inducing *Pten* excisions in the developing prostate. While the progression through the various disease stages is not as rapid as that seen in the *PBCre4×Pten^flox/flox^* mice, we did see an accelerated phenotype as compared to that observed when *Pten* was excised in the fully developed prostate. In summary, our results suggest that the tumor promoting effects of *Pten* deficiency are highly dependent on the developmental stage of the prostate.

## Materials and Methods

### Genotyping

*ARR2PB-CreER(T2)* mice [21] were genotyped using the following primers to verify for germline transmission: HPRT-FP: cct tac ctt tca gaa gta ggc and CreER(T2)2: atc aac gtt ttc ttt tcg g. *Pten^flox/flox(fl/fl)^* mice were genotyped with Pten Flox-1: ctc ctc tac tcc att ctt ccc and Pten Flox-2: act ccc acc aat gaa caa ac.

### In vivo experiments

Animals (mixed 129;C57BL/6 background) were injected with OHT at 6 wks of age, a time when stereological evaluation reveals the prostate to be fully developed [31] and at 2 wks of age, a time when prostate development is just commencing [31]. *ARR2PBCreER(T2)×Pten^fl/fl^* mice and control mice lacking the Cre transgene were injected i.p. with 0.1 mg OHT in corn oil vehicle for five consecutive days and then allowed to age for the following periods: 4–10, 16–20, 30–40, and 42–52 wks for the mice injected at 6 wks of age; and for 4–6 and 10–12 wks post-injection (p.i.) for mice injected with OHT at 2 wks of age. Furthermore, a cohort of non-OHT-exposed *ARR2PBCreER(T2)×Pten^fl/fl^*, *Pten^fl/fl^* and *ARR2PBCreER(T2)* mice were left to age for 16–20 wks to control for the possibility of spontaneous *Pten* excisions. Mice were housed in a viral antibody-free barrier facility in accordance with Canadian Council on Animal Care, and the University of Calgary Animal Care committee guidelines.

### Histology and Immunohistochemistry

The dissected lower urogenital tracts of the mice were fixed in 10% formaldehyde, embedded in paraffin and sectioned at 5 µm. Sections were de-paraffinized with xylene and graded alcohols and every 10^th^ section was stained with hematoxylin and eosin (H&E) for microscopy. For immunohistochemistry, sections were de-paraffinized as above and antigen retrieval was performed using 10 mM sodium citrate, pH 6.0. Antibodies used were mouse anti-Pten antibody (1∶120); mouse anti-phospho-AKT (1∶200); rabbit anti-phospho-mTOR (1∶7); rabbit anti-phospho-4E-BP1 (1∶5); rabbit anti-eIF4E antibody (1∶10); rabbit anti-phospho-S6 ribosomal protein (Ser 240/244) antibody (1∶100) (all from Cell Signaling). Anti-high molecular weight cytokeratin antibody clone 34BE12 (1∶100) was from Dako; and either the Vectastain Elite mouse IgG kit or the rabbit IgG ABC kit from Vector Laboratories were used as the secondary reagents. Color development was performed with DAB substrate (Sigma-Aldrich) and sections were counterstained with hematoxylin.

### Apoptosis assay

The dissected lower urogenital tracts of the mice were fixed in 10% formaldehyde, embedded in paraffin, and sectioned at 5 µm. Sections were de-paraffinized with xylene and graded alcohols prior to the TUNEL assay, which was performed with the ApopTag peroxidase *in situ* apoptosis detection kit according to the manufacturer's instruction (Chemicon International, Inc., Temecula, CA).

### Quantification of proliferation and apoptosis

The proliferation index was determined as the percentage of Ki67-positive cells over total cells counted. At least 500 cells per animal were counted over 5 different fields of view and 3–4 mice were included per group. Quantification of apoptosis was performed in a similar manner by determining the percentage of TUNEL positive cells over total number of cells, except that 1000 cells were counted per animal (n = 3–4 mice per group). Student T-Test (two-tailed; unequal variance) was used for statistical analyses.
